# Supplementary material for: Ultrafast melting and recovery of collective order in the excitonic insulator Ta2NiSe5
Source: Nat Commun. 2021 Mar 16;12:1699. doi: 10.1038/s41467-021-21929-3 (PMC7966769; doi:10.1038/s41467-021-21929-3)
Supplement: Supplementary file 1 — Supplementary Information [file 41467_2021_21929_MOESM1_ESM.pdf]

Supplementary Information for:

Ultrafast melting and recovery of collective order in the excitonic insulator Ta_2NiSe_5

Hope M. Bretscher[1,†], Paolo Andrich[1,†,*], Prachi Telang[2], Anupam Singh[2], Luminita Harnagea[2], A. K. Sood[3], and Akshay Rao[1,*]

[1]*Cavendish Laboratory, University of Cambridge, Cambridge CB3 0HE, United Kingdom*

[2]*Department of Physics, Indian Institute of Science Education and Research, Pune, Maharashtra 411008, India*

[3]*Department of Physics, Indian Institute of Science, Bangalore, Karnataka 560012, India*

[†] These authors contributed equally

[*] Correspondence should be addressed to P.A. (pa343@cam.ac.uk) or A. R. (ar525@cam.ac.uk)

In these supplementary notes, we report additional details of the experimental methods and data analysis introduced in the main text.

In Note 1, we present the effect of the probe polarisation on the $\Delta R/R$ signal. Additionally, we show the temperature dependent data collected using probe pulses polarised orthogonal to the Ta and Ni atomic chains (along the c axis, see Fig.1b in the main text).

In Note 2, we discuss the model used to fit the kinetics of the signal in the NIR wavelength range.

In Note 3, we report the temperature dependence of the signal amplitude at wavelengths different from that used in the main text.

In Note 4, we describe how time zero is determined in our measurements and show the details of the $\Delta R/R$ signal around this time delay.

In Note 5, we present the calculation of the out-of-equilibrium optical conductivity of the material to analyse the change in spectral weight at 1200 nm in the presence of strong photoexcitation or high temperatures.

In Note 6, we investigate the effect of temperature on the threshold fluence required to melt the excitonic order.

In Note 7, we discuss in more detail the behaviour of the $\Delta(\Delta R/R)$ signal amplitude as a function of Φ_{P1}.

In Note 8, we present the temperature and fluence dependence of the 1 THz phonon mode deduced from two-pulse measurements.

Note 1 – Effect of probe polarisation

The transient reflectivity signal of Ta_2NiSe_5 in the visible and near infra-red (NIR) region strongly depends on the orientation of the probe polarisation, as it is expected from equilibrium optical measurements[1,2]. Supplementary Fig. 1a shows the *ΔR/R* spectra (averaged between 0.5 and 1 ps) collected for probe polarisations along the *a* and *c* axis at room temperature and using a pump fluence Φ_{pump} = 45 μJ/cm^2. We attribute this drastic difference to the strong 1D nature of Ta_2NiSe_5, which implies that different probe polarisation are sensitive to different electronic transitions.

In Supplementary Fig. 1b we present the evolution of the transient reflectivity spectra as a function of temperature for a probe polarisation along the c axis. The signal in the NIR shows a strong temperature dependence but, contrary to the behaviour presented in Fig. 1b of the main text, it does not appear to saturate as the temperature rises above T_c. Remarkably, the signal in the visible range shows no dependence on the temperature. Further studies are required to determine the nature of the electronic transitions involved in the material's optical response and their relationship to its electronic properties.

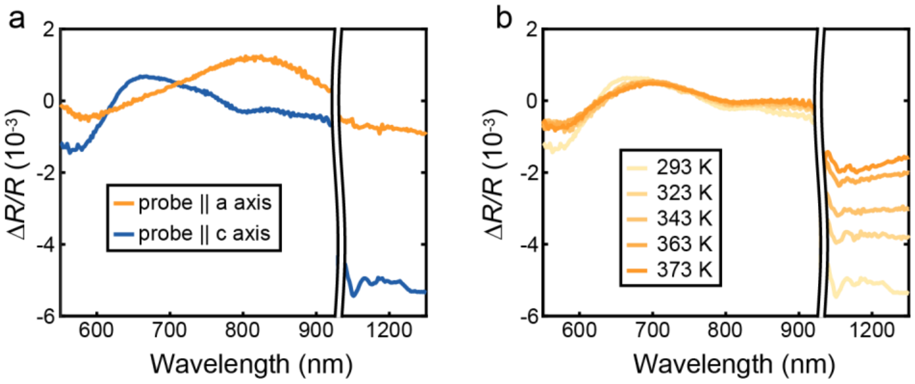

Supplementary Fig. 1. Polarisation dependent *ΔR/R* (a) Comparison of the *ΔR/R* signal for a probe polarisation oriented along the *a* (orange) and *c* (blue) axis of the Ta_2NiSe_5 crystal. (b) Spectra of the *ΔR/R* signal collected with a probe polarisation along the *c* axis for a series of temperatures.

Note 2 – Fitting model

The fit of the electronic (incoherent) component of the signal for all temperatures and fluences is hindered by the drastic change in the kinetics as a function of these physical quantities. For this reason we are not able to provide a complete quantitative analysis of all fitting parameters. We focus instead only on the value of the signal amplitude, whose behaviour is not influenced

by the specifics of the fitting function used. In addition, we employ this fitting procedure to isolate the coherent component of the signal associated to the phonon oscillations.

We consider a functional form commonly used to describe the transient optical response in condensed matter systems[3,4]:

$$\frac{\Delta R(t)}{R} = \left\{\frac{A_1}{2} \times \left[\mathrm{erf}\left(\frac{t-t_0}{\tau_{r,1}}\right)+1\right] \times \left[\exp\left(-\frac{t-t_0}{\tau_d}\right)\right]\right\} + \left\{\frac{A_2}{2} \times \left[\mathrm{erf}\left(\frac{t-t_0}{\tau_{r,2}}\right)+1\right]\right\} \quad (1)$$

The terms in curly brackets are associated to the rise and decay of the signal at early times (< 2 ps) and to the slow signal growth at long times (> 2 ps) respectively. In particular, A_1 and A_2 are the amplitudes of these two components, the error functions describe the rise of the signal with characteristic time $\tau_{r,1}$ and $\tau_{r,2}$, and the exponential term captures the signal decay with characteristic time τ_d.

In Supplementary Fig. 2, we show a subset of the kinetics traces from Fig.1c with the relative fit obtained using Supplementary Eq. (1).

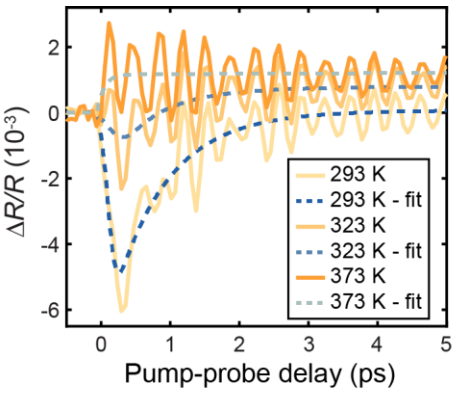

Supplementary Fig. 2. Fits of incoherent signal. Kinetic of the *ΔR/R* signal at a probe wavelength of 1200 nm (orange solid lines), as in Fig. 1c. The dashed lines are the fit to the data calculated using Supplementary Eq. (1).

Note 3 – Wavelength dependence of temperature behaviour

In Supplementary Fig. 3 we show the dependence of A_1 as a function of temperature (in analogy with the plot in Fig. 1d of the main text) for the 800 nm component of the probe pulse. The behaviour is the same previously observed in other works[4,5] and is different from that observed in the NIR region. This underscores the uniqueness of the wavelength region investigated in our work, which allows us to easily follow the evolution of the excitonic order.

In Supplementary Fig. 4 we present the dependence of A_1 as a function of temperature for two additional wavelengths in the NIR. As mentioned in the main text, the precise temperature at which the signal switches sign shifts slightly as a function of the probe wavelength, but the

overall behaviour is consistent across the NIR range detected in our measurements. We note that the dashed line is obtained with the same equation used in the main text, in particular using the same value for T_c = 328 K.

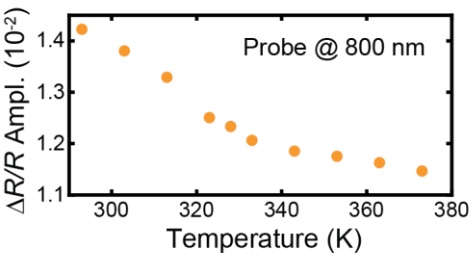

Supplementary Fig. 3. Amplitude of the *ΔR/R* signal as a function of temperature for a 800 nm probe wavelength. The observed behaviour is analogous to that reported in Fig. 3a of ref. 5.

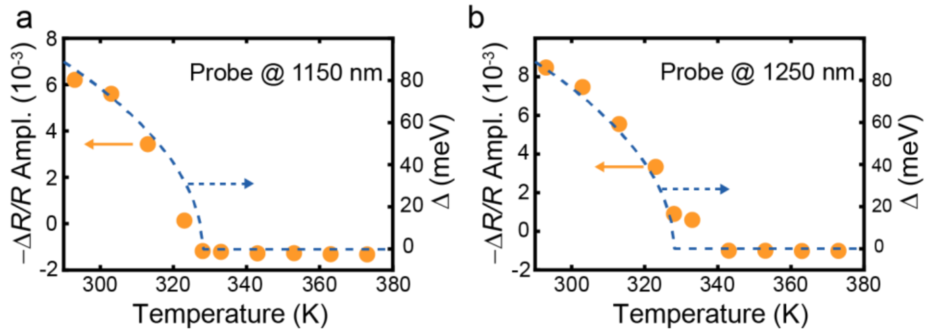

Supplementary Fig. 4. Order parameter behaviour at various wavelengths. Amplitude of the *ΔR/R* signal as a function of temperature (orange dots, left y-axis) in correspondence of a probe wavelength of (a) 1150 nm and (b) 1250 nm. As in the main text the blue dashed line (right y-axis) represents the temperature dependence of a BCS-like order parameter calculated using[6] $\Delta(T)/\Delta(0) \approx 1.74(1 - T/T_c)^{0.5}$, with T_c= 328 K.

Note 4 – Time zero, coherent artefact and free-induction decay

In our broadband measurements the probe pulse reflected by the sample is collected and dispersed onto a CCD camera to achieve spectral resolution. As the pulse is naturally chirped each spectral component arrives at the detector at slightly different times. In particular, in our measurements, the longest wavelengths are at the front of the probe pulse while the shorter ones trail.

For what concerns the pump-probe measurements, we define time zero as the probe delay for which the transient reflectivity signal (after the coherent artefact, see below) becomes visible at the shorter wavelengths, that is when the pump pulse “catches up” with the tail of the probe pulse. The signal is then chirp-corrected in the data analysis stage to report all the wavelength components with their correct time zero, as it is commonly done in broadband pump-probe

spectroscopy[7]. In the three-pulse experiments we find time zero by performing the same procedure on P1 and P2 separately.

We note that the transient reflectivity is not identically zero before a time zero determined as described above. As shown in Supplementary Fig. 5, the signal instead shows oscillations that are particularly visible at the higher excitation fluences. The largest feature right before time zero is the so-called coherent artefact occurring when pump and probe pulses overlap in space and time in the material[8]. The smaller oscillations are also a well-known effect, arising particularly in experiments that use ultra-short excitation pulses. It is typically referred to as perturbed free-induction decay and does not carry any obvious information on the material[9]. When we focus on the analysis of the $\Delta R/R$ signal at early times we cut out this “signal” before time zero to simplify the understanding of our discussion.

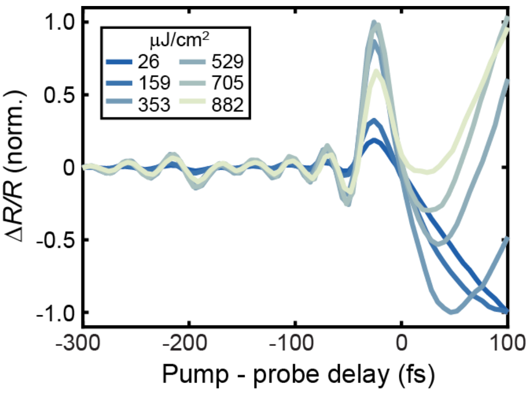

Supplementary Fig. 5. Signal rise as a function of fluence. Normalised $\Delta(\Delta R/R)$ signal (analogous to that reported in Fig. 2b) at short probe delays and before time zero.

Note 5 – Time-resolved evolution of the optical conductivity

In the main text, we explain that the peculiarity of the spectral region around 1200 nm (1.03 eV) might be associated to a shift in the spectral weight of the system’s electronic as the excitonic condensate is melted. This interpretation is substantiated by previously published work on the temperature dependence of the real part of the optical conductivity (σ_1) of Ta_2NiSe_5[10]. In the equilibrium data reported there, a clear feature centred around 1.05 eV (that we refer to as F_{1eV} in the following) is observed below T_c, while it is completely suppressed at high temperatures. The spectral weight associate to this feature could then be connected to electronic states only available when the excitonic gap is open (and therefore spectral weight is shifted up from the low energy scale).

To shed more light on this physical process and to support our conclusions, we used the equilibrium data (that the group of Professor A.V. Boris at the Max Planck Institute for Solid State Research kindly shared with us) together with our pump-probe signal to reconstruct the time evolution of σ_1. In particular, we compare how this quantity evolves after the material is

perturbed at room temperature in the presence of a weak and strong pump pulse, and at a temperature above T_c.

As mentioned above, we begin with the equilibrium σ_1 at the temperature of interest as measured in [10]. Using a Kramers-Kronig constrained variational analysis[11], we can then extract the equilibrium reflectivity ($R_0(\omega)$) of the sample. Turning now to our pump-probe measurements, the signal that we collect is $\Delta R(\omega,t)/R_0(\omega) = R(\omega,t)/R_0(\omega) - 1$. Using the calculated $R_0(\omega)$, we can therefore obtain the reflectivity of the material $R(\omega,t)$ for every value of the pump-probe delay. Finally, using a differential Kramers-Kronig constrained variational analysis step[12], we can calculate the time dependent optical conductivity $\sigma_1(\omega,t)$.

In Supplementary Fig. 6, we report the result of this analysis applied to the room temperature pump-probe data with $\Phi_{\text{pump}} = 26$ μJ/cm^2. In Supplementary Fig. 6(a), we plot σ_1 over the energy range between 0.9 eV and 1.7 eV. Here, we see that the region around F_{1eV} shows the largest time dependent change in these conditions. In Supplementary Fig. 6(b) we focus on this region to inspect the effect of the pump perturbation more closely. At this low fluence, F_{1eV} clearly exists at all times in the optical conductivity. The feature is marginally suppressed immediately after photoexcitation and returns to the equilibrium condition at long delays. This is the behaviour that one would expect in the presence of a perturbation process that partially suppresses the excitonic order.

In Supplementary Fig. 7(a,b) we show the result of the same calculation for the case of T = 343 K (the highest temperature for which the equilibrium data is available) and $\Phi_{\text{pump}} = 176$ μJ/cm^2. We choose to focus on a higher fluence than in the room temperature case because the *ΔR/R* signal is much smaller above T_c. Here, we see a very different behaviour for σ_1. As we mentioned, the equilibrium optical conductivity does not include F_{1eV} at this temperature. The time dependent $\sigma_1$increases after photoexcitation uniformly across the whole energy range, a behaviour compatible with the temporary enhancement of a Drude component to σ_1 induced by the excitation of quasi-particles across the energy gap. This appears to be the strongest contribution above T_c.

We now have all the necessary elements to analyse the material's response at room temperature and in the presence of a high excitation fluence (see Supplementary Fig. 7(c,d) for the case of $\Phi_{\text{pump}} = 705$ μJ/cm^2). In this case we see that $\sigma_1$very rapidly evolves to resemble the high temperature response. In particular, F_{1eV} is completely suppressed within ~ 125 fs and the response is dominated by a Drude-like increasing $\sigma_1$at pump-probe delays up to ~ 1.5 ps. At even longer delays we witness a recovery of F_{1eV} and σ_1 progressively approaches the equilibrium curve, with a slightly more pronounced tail at higher energies that could be associated to the onset of a bolometric effect.

The results presented here support our conclusion that the optical response of Ta_2NiSe_5 at 1200 nm mirrors the low-energy physics of this system. In particular, we demonstrate that, in the presence of a strong photoexcitation, the transient optical conductivity becomes analogous to the equilibrium one at temperatures above T_c. We can therefore use the *ΔR/R* signal to track the status of the excitonic condensate as explained in the main text. We stress that this is not

the only evidence of the photoinduced phase transition. The measured phononic response provides another very important contribution to our analysis.

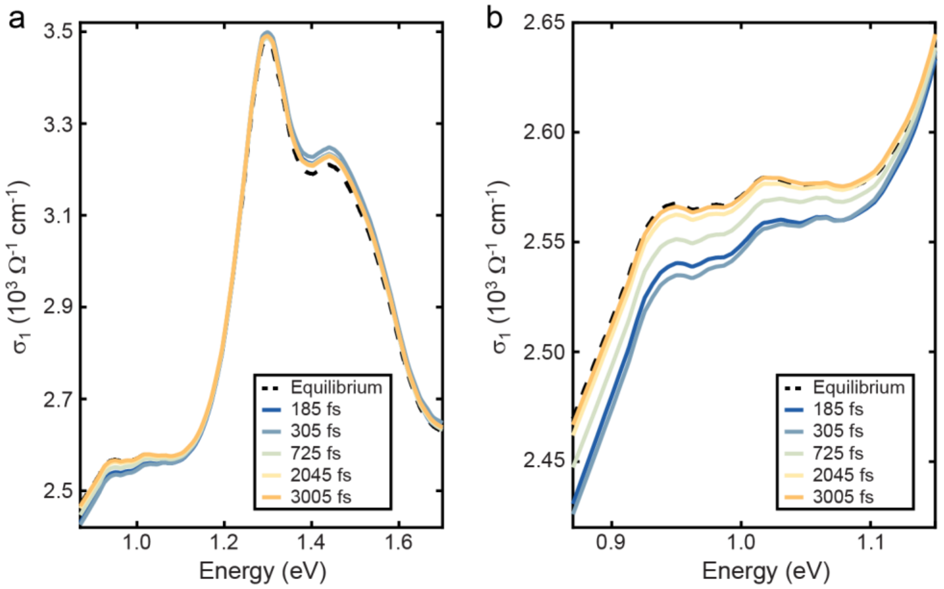

Supplementary Fig. 6. Optical conductivity at room temperature and low-fluence. Evolution of σ_1 after a pump pulse of fluence Φ_{pump} = 26 µJ/cm^2 at room temperature (a) in the range between 0.9 and 1.7 eV (730 – 1380 nm) and (b) around F_{1eV}. The dashed curve represents σ_1 in equilibrium (as measured in [10]), while the solid traces show its value at different pump-probe delays.

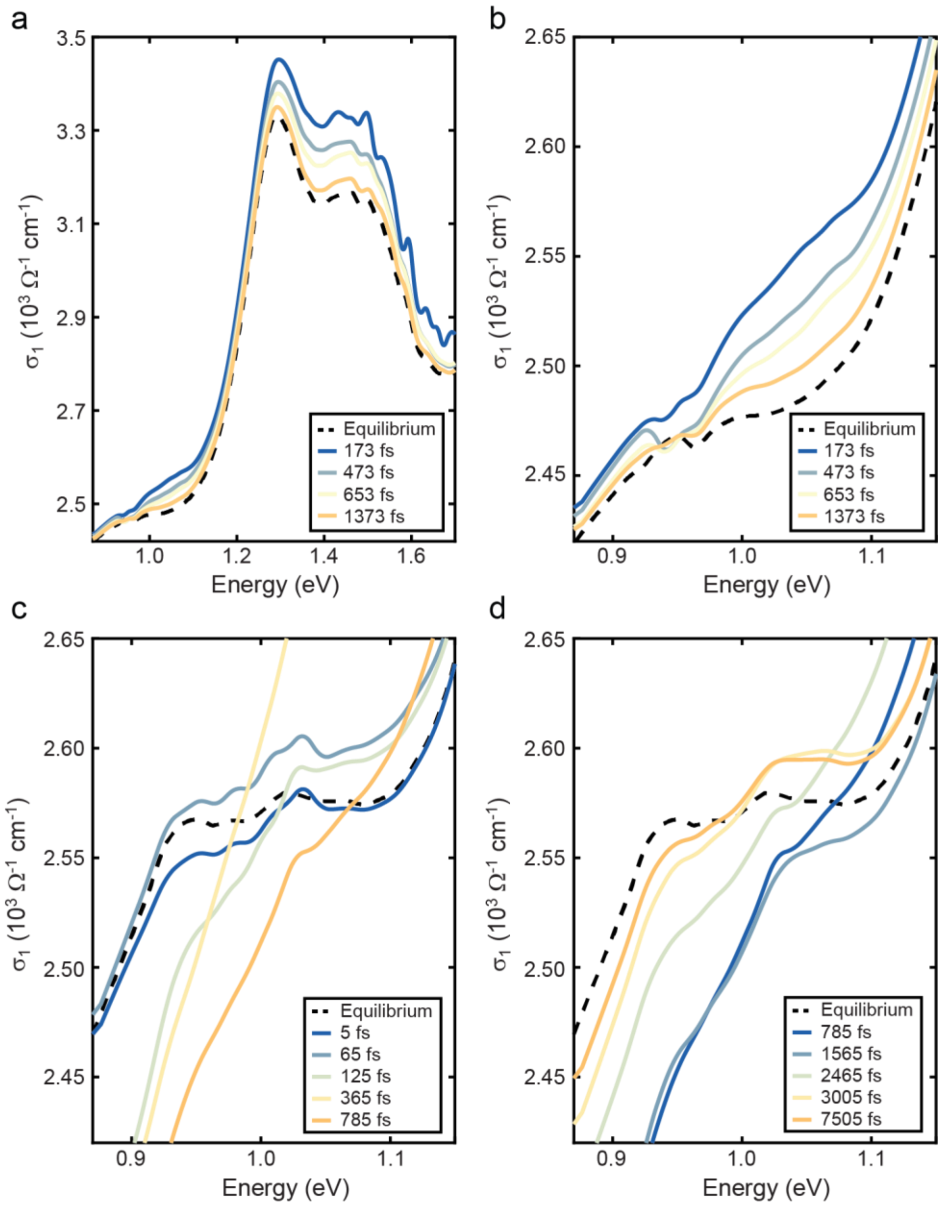

Supplementary Fig. 7. Optical conductivity comparing high temperature to room-temperature, high fluence. (a,b) Evolution of σ_1 after a pump pulse of fluence Φ_{pump} = 176 μJ/cm^2 at 343 K in the 0.9 – 1.7 eV range (a) and around F_{1eV} (b). (c,d) Evolution of σ_1 after a pump pulse of fluence Φ_{pump} = 705 μJ/cm^2 at room temperature for short (c) and long (d) pump-probe delays. The dashed curve represents the equilibrium σ_1 at 343 K (a,b) and at room temperature (c,d). The solid traces show σ_1 at different pump-probe delays.

Note 6 – Temperature dependence of the photoinduced melting threshold

One consequence of our interpretation of the data in Fig. 2 is that we expect the pump fluence required to melt the excitonic order to become smaller as we approach T_c. This indeed simply reflects the effect of the temperature on the amplitude of the excitonic order parameter (or alternatively of the EI gap). To demonstrate that this is the case, in Supplementary Fig. 6 we report the time traces at two fixed pump fluences for a few different sample temperatures. Here,

we see that pump fluences that are not sufficient to photoinduce a phase transition at room temperatures reside instead above this threshold fluence when we move closer to T_c, as expected. For the sake of completeness, we also show the data collected above T_c.

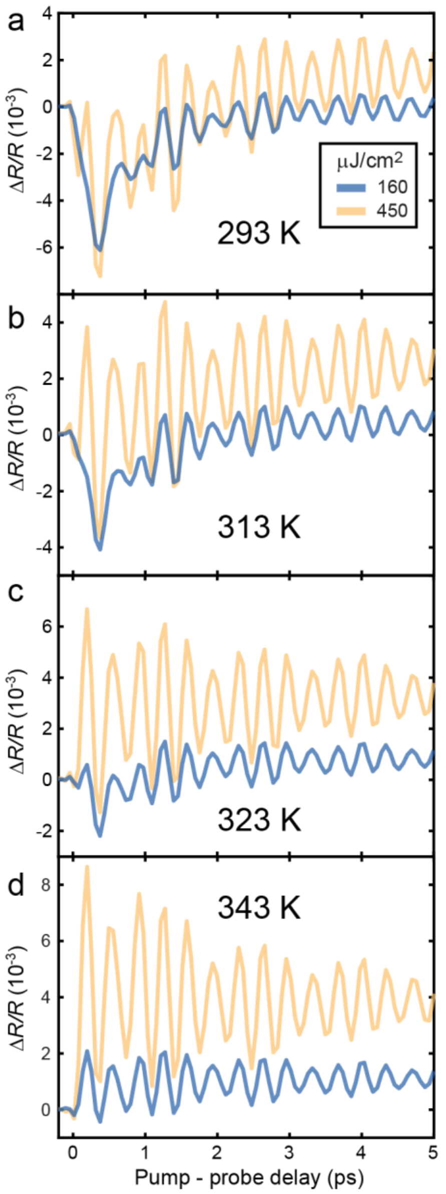

Supplementary Fig. 8. Temperature-dependent critical fluence. *ΔR/R* kinetic at 1200 nm for two values of Φ_{pump} at temperatures (a) 293 K, (b) 313 K, (c) 323 K, and (d) 343 K.

Note 7 – Decay of the signal amplitude in the multi-pulse scheme

In Supplementary Fig. 4 we report the data of Fig. 3d normalised to enable a comparison of the signal recovery time τ_{cond}. Here we see that, while no clear increase in τ_{cond} is observed for the lower values of Φ_{P1}, a slower relaxation process becomes obvious for Φ_{P1} = 882 μJ/cm^2. We attribute this to the strong suppression of the excitonic condensate at high excitation fluences, which results in the transition of the system to an insulating phase of uncorrelated excitons

(pre-formed exciton phase). The slower recovery could also be associated to the presence of topological defects separating regions with non-zero order parameter, as previously observed in other systems[13–16].

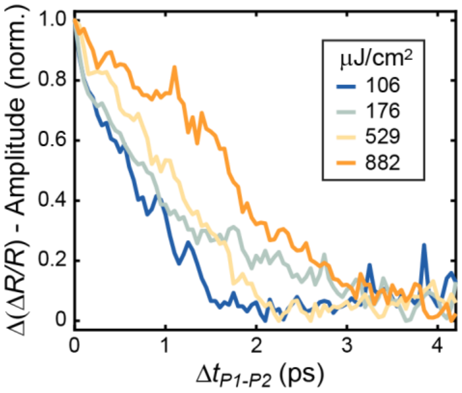

Supplementary Fig. 9. Fluence-dependent decay amplitude. Evolution of the normalised *Δ(ΔR/R)* amplitude as a function of the P1 – P2 delay for different values of Φ_{P1}.

Note 8 – Temperature and fluence dependence of 1 THz phonon mode

Supplementary Fig. 5 summarises the effect of temperature and excitation fluence on the amplitude of the 1 THz phonon mode ($A_{ph,1}$) extracted using the two-pulse technique. While at the lowest fluence $A_{ph,1THz}$ is independent from the sample temperature, we observe that under stronger photoexcitation a saturation is reached much faster at high temperatures. This data complements the results described in Fig. 4 of the main text and supports the claim that the behaviour of the 1 THz phonon is intimately connected with the material state. At the same time, this observation calls for a reassessment of the data interpretation advanced in previous works[4].

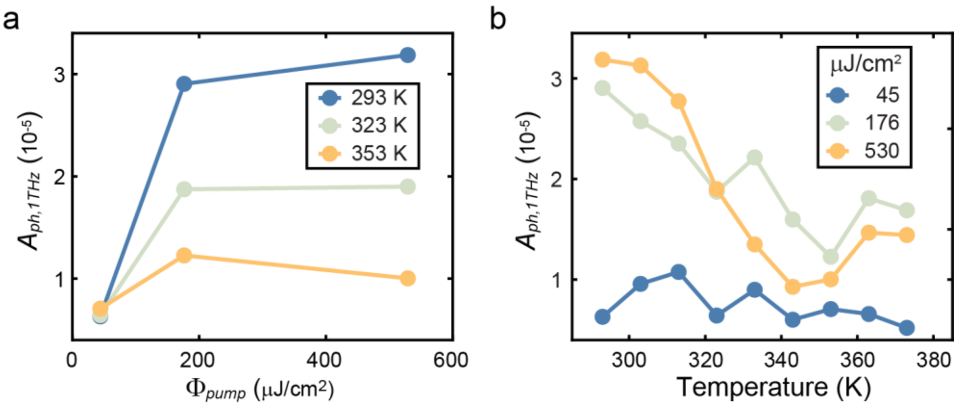

Supplementary Fig. 10. Saturation behaviour of 1 THz phonon mode. Amplitude of the 1 THz phonon mode measured with the two-pulse pump-probe technique (a) as a function of pump fluence for a series of temperatures and (b) as a function of temperature for three different pump fluences.

1. Larkin, T. I. Excitonic Fano resonances in Ta_2NiSe_5 and Ta_2NiS_5. *Online Publ. der Univ. Stuttgart* (2016).

2. Seo, Y.-S. *et al.* Temperature-dependent excitonic superfluid plasma frequency evolution in an excitonic insulator, Ta_2NiSe_5. *Sci. Rep.* **8**, 11961 (2018).

3. Demsar, J., Sarrao, J. L. & Taylor, A. J. Dynamics of photoexcited quasiparticles in heavy electron compounds. *J. Phys. Condens. Matter* **18**, R281–R314 (2006).

4. Werdehausen, D. *et al.* Coherent order parameter oscillations in the ground state of the excitonic insulator Ta_2NiSe_5. *Sci. Adv.* **4**, eaap8652 (2018).

5. Werdehausen, D. *et al.* Photo-excited dynamics in the excitonic insulator Ta_2NiSe_5. *J. Phys. Condens. Matter* **30**, 305602 (2018).

6. Tinkham, M. *Introduction to superconductivity*. (McGraw-Hill, 1980).

7. Megerle, U., Pugliesi, I., Schriever, C., Sailer, C. F. & Riedle, E. Sub-50 fs broadband absorption spectroscopy with tunable excitation: putting the analysis of ultrafast molecular dynamics on solid ground. *Appl. Phys. B Lasers Opt.* **96**, 215–231 (2009).

8. Lebedev, M. V., Misochko, O. V., Dekorsy, T. & Georgiev, N. On the nature of 'coherent artifact'. *J. Exp. Theor. Phys.* **100**, 272–282 (2005).

9. Singla, R. *et al.* Photoinduced melting of the orbital order in La0.5Sr 1.5MnO4 measured with 4-fs laser pulses. *Phys. Rev. B - Condens. Matter Mater. Phys.* **88**, 2–5 (2013).

10. Larkin, T. I. *et al.* Giant exciton Fano resonance in quasi-one-dimensional Ta_2NiSe_5. *Phys. Rev. B* **95**, 195144 (2017).

11. Kuzmenko, A. B. Kramers-Kronig constrained variational analysis of optical spectra. *Rev. Sci. Instrum.* **76**, 1–9 (2005).

12. Novelli, F. *et al.* Ultrafast optical spectroscopy of the lowest energy excitations in the

Mott insulator compound YVO 3: Evidence for Hubbard-type excitons. *Phys. Rev. B - Condens. Matter Mater. Phys.* **86**, 2–7 (2012).

13. Yusupov, R. *et al.* Coherent dynamics of macroscopic electronic order through a symmetry breaking transition. *Nat. Phys.* **6**, 681–684 (2010).

14. Lee, W. S. *et al.* Phase fluctuations and the absence of topological defects in a photo-excited charge-ordered nickelate. *Nat. Commun.* **3**, 838 (2012).

15. Mertelj, T. *et al.* Incoherent Topological Defect Recombination Dynamics in $TbTe_3$. *Phys. Rev. Lett.* **110**, 156401 (2013).

16. Zong, A. *et al.* Evidence for topological defects in a photoinduced phase transition. *Nat. Phys.* **15**, 27–31 (2019).
