# Supplementary material for: Ultrafast melting and recovery of collective order in the excitonic insulator Ta2NiSe5
Source: Nat Commun. 2021 Mar 16;12:1699. doi: 10.1038/s41467-021-21929-3 (PMC7966769; doi:10.1038/s41467-021-21929-3)
Supplement: Supplementary file 2 — Peer Review File [file 41467_2021_21929_MOESM2_ESM.pdf]

Editorial Note: This manuscript has been previously reviewed at another journal that is not operating a transparent peer review scheme. The manuscript was considered suitable for publication without further review at *Nature Communications.*
